# Electrifying the UNSDGs with microbial power

**DOI:** 10.1042/ETLS20253010

**Published:** 2025-12-08

**Authors:** Sai Kushal Kumar Solleti, Sahashransu Satyajeet Mahapatra, S. K. Shakthi Thangavel, A. S. Vishwanathan

**Affiliations:** 1 WATER Laboratory, Department of Biosciences, Sri Sathya Sai Institute of Higher Learning, Prasanthi Nilayam, Puttaparthi, Andhra Pradesh, 515134, India

**Keywords:** electrogenic bacteria, microbial biotechnology, microbial electrochemical systems, sustainable development goals, water scarcity

## Abstract

Water stress represents a critical global challenge demanding innovative solutions for effective water resource management. Microbial electrochemical technologies (METs) leverage bacterial extracellular electron transfer for addressing issues related to water stress. These technologies exhibit a diverse range of applications, positioning them as integral to sustainable development through effective water resource management. Their versatility allows them to function as key contributors to achieving the United Nations Sustainable Development Goals (UNSDGs). Through their application, METs offer promising strategies for mitigating pollution, recovering valuable resources, and enabling real-time water quality monitoring. Employing these technologies facilitates the concurrent addressing of various UNSDGs, fostering a holistic and integrated approach. METs present opportunities for decentralized wastewater treatment and reuse, thereby promoting accessibility to clean water and sanitation, particularly in marginalized communities. However, the realization of these benefits faces significant challenges, including technological scalability, optimization, and regulatory frameworks. Overcoming these obstacles is crucial for harnessing the full potential of METs to meet UNSDGs. This perspective article underscores the imperative of further research, collaboration, and policy support to propel METs towards becoming a cornerstone in the sustainable management of water resources and the achievement of UNSDGs on a global scale.

## The United Nations Sustainable Development Goals

**Figure 1:**
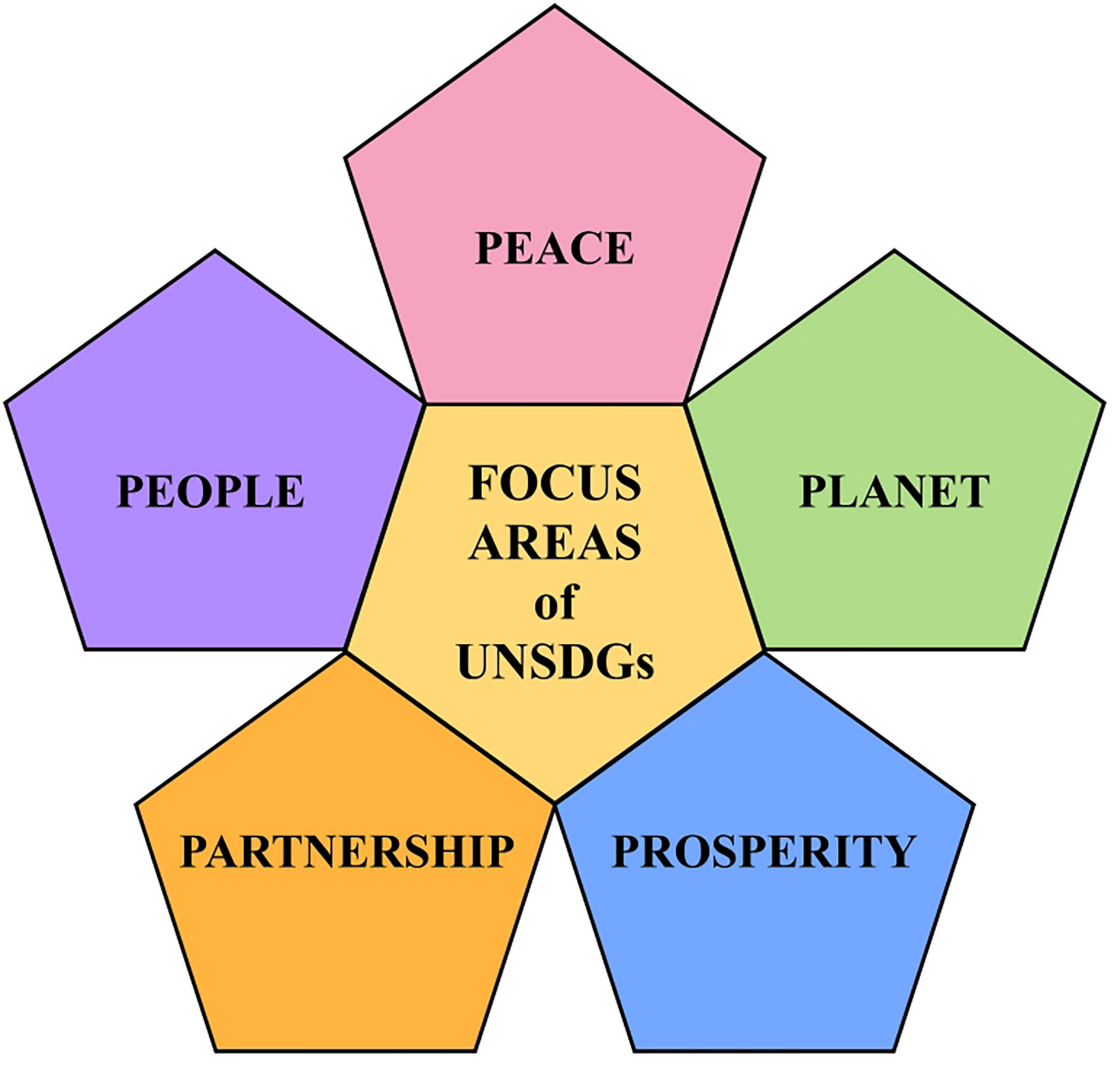
The five critical areas of the United Nations Sustainable Development Goals.

The United Nations General Assembly, with unanimous support from 193 countries and numerous non-governmental organizations (NGOs), adopted the United Nations Sustainable Development Goals (UNSDGs) in 2015, building upon the Millennium Development Goals. The 2030 Agenda for Sustainable Development [1] describes these 17 goals and 169 targets as integrated and indivisible, balancing the economic, social, and environmental dimensions of sustainable development. These ambitious goals, aimed at advancing living standards and addressing global issues, are anchored in five areas of critical importance known as the ‘5 Ps’ (Figure 1). The agenda [1] is fundamentally about ‘people,’ pledging to end poverty and hunger while ensuring all human beings can fulfill their potential in dignity and equality. This is intrinsically linked to the ‘planet,’ with a commitment to protect Earth’s natural resources, biodiversity, and climate for future generations. The goals aim for ‘prosperity,’ ensuring that all people can enjoy fulfilling lives through economic and social progress that occurs in harmony with nature. This vision is built upon a foundation of ‘peace,’ recognizing that sustainable development requires fostering peaceful, just, and inclusive societies free from fear and violence. Finally, achieving these multifaceted goals necessitates a robust ‘partnership,’ revitalizing the global partnership for sustainable development through a spirit of strengthened solidarity and co-operation among all countries and stakeholders.

### Current status of progress

Despite the initial commitment and efforts, the 2025 Sustainable Development Goals Report [2]⁠ indicates that progress towards achieving the UNSDGs has been slow and uneven. The assessment of the sustainable developmental goal(SDG) targets reveals that progress is largely insufficient, with only 35% of them on track or making moderate gains. Most concerningly, 48% show insufficient or marginal progress, and 18% have regressed below the 2015 baseline. Large-scale disruptions like the COVID-19 pandemic, alongside persistent challenges such as climate change and geopolitical conflicts, have impacted economic stability and impeded progress, disproportionately affecting poor and vulnerable communities. While developed nations have shown leadership in meeting many targets, significant disparities remain when compared with less-privileged nations. A literature review [3] suggests that while economy-related targets were generally on track, those related to education and climate change required greater impetus. The halfway review summit in 2023 called for renewed commitment [4]. The UN Statistics Division, in partnership with Google, launched the ‘UN Data Commons for the SDGs’ platform [5] which ‘integrates authoritative SDG data and insights from across the UN system into a public repository with advanced search functionality and an innovative, user-friendly interface,’ making authenticated datasets available ‘to ultimately facilitate informed data-driven decisions.’

Getting the UNSDGs back on track necessitates concerted efforts from both developed and developing nations. Prioritizing the understanding of interlinkages between the UNSDGs is crucial for channeling efforts effectively [6]. Partnerships involving governmental and non-governmental entities can leverage unique strengths to address complex challenges. Aligning national development frameworks with UNSDGs, coupled with periodic reviews, is essential for adaptation, adoption, and progress assessment. Bridging the gap between developed and developing nations requires the mobilization of financial resources and the transfer of technologies. Strengthening data collection and monitoring mechanisms is also critical for accurate progress tracking, recognizing that ‘what is not measured, cannot be improved.’ Identifying and resolving the root causes of impediments is necessary to prevent recurrence. Stakeholders must be held accountable for their commitments. Sustainable development education must be incorporated in school and university curricula to increase awareness about the UNSDGs and instill a sense of responsibility among students [7]. Furthermore, adopting a diverse, systematic, and innovative approach to augment the SDG indicator framework has been suggested [8].

The achievement of the UNSDGs is inextricably linked to the availability and sustainable management of water resources. Water scarcity and pollution significantly impact human health, food security, and economic development, thereby hindering progress across multiple SDGs [9]. Water is a rapidly depleting natural resource, with demand increasing by ∼1% annually over the last 40 years, and this trend is projected to continue until 2050. More than half the world’s population experiences extreme water stress for at least a month each year. The impact of water stress is more intense in lower- and middle-income countries, particularly in emerging economies [4]. This pressing need for efficient water management has driven the development of various physical, chemical, and biological approaches for repurposing wastewater as a resource to reduce water stress.

## Microbial Electrochemical Technologies

Microbial fuel cells are devices that generate a flow of electrons catalyzed by electrogenic bacteria. Their architecture spatially segregates substrate oxidation and oxygen reduction for effective electron capture and utilization. Substrate oxidation typically occurs under anoxic conditions to facilitate electron transfer from bacteria to an electrically conductive anode. The flow of electrons across a wired circuit to a cathodic terminal electron acceptor (often oxygen) can be diverted for various applications. Focused research on bacterial electron transfer has spurred the development of diverse microbial electrochemical technologies (METs). These technologies represent engineering variations centered around the theme of electron transfer to and from electrodes by electrogens and electrotrophs, respectively. METs, combining biological and electrochemical processes, leverage the metabolic activities of microorganisms to remediate environmental pollutants, generate electricity, and produce valuable chemicals [10].

At the core of METs lies extracellular electron transfer, a bioelectrochemical process where electrochemically active bacteria transfer electrons across their cell membrane to external acceptors such as electrodes, enabling the conversion of chemical energy in organic matter to electrical current or driving reduction reactions. Exoelectrogenic bacteria, which specifically release electrons extracellularly, comprise a broad range of bacterial species, also including archaea and actinobacteria [11]. This transfer of electrons can occur directly via outer membrane cytochromes, through conductive extensions known as pili, or by using endogenous shuttles [12]. Non-exoelectrogenic bacteria can also participate with the aid of externally supplied mediator molecules. Under stress, bacteria aggregate to form biofilms that are complex, dynamic microhabitats supporting the bacterial community [13]. Biofilms constituted by electroactive bacteria within a conductive extracellular matrix possess the additional capacity to exchange electrons. These electroactive biofilms can be viewed as ‘intelligent’ systems capable of storing electrons and regulating their flow efficiently [14]. Some bacteria perform extracellular electron uptake, reducing extracellular electron donors while moving electrons into the cell [15].

Specific applications of METs include, but are not limited to:

- Sediment microbial fuel cells, which exploit microbial communities in organic-rich sediments for environmental remediation and energy harvesting to power underwater sensors [16].
- Photosynthetic microbial fuel cells, which utilize phototrophic organisms as oxygenators at the cathode, enabling sustainable electricity production alongside nitrogen and phosphorus removal [17].
- Microbial electrolysis cells, which are primarily used for hydrogen production from water and breaking down various toxic compounds, making them applicable in environmental remediation and as water quality sensors [18].
- Microbial desalination cells, which use electrons from exoelectrogens coupled with external voltage to drive ion transport through membranes for seawater desalination [19].
- Microbial electrosynthesis cells, which utilize electrotrophs to take up electrons from an electrode to convert CO_2_ into valuable products like short-chain fatty acids and alcohols for biodiesel production [20].
- Beyond standalone systems, microbial fuel cells have been integrated with conventional processes, such as constructed wetlands [21], anaerobic digesters [22], and membrane bioreactors [23], to enhance performance.

### Harnessing METs for sustainable environmental management

METs enable the design of modular, decentralized wastewater treatment systems suitable for remote and resource-limited areas where centralized infrastructure is economically unfeasible. The integration of METs with conventional water treatment processes holds the promise of enhanced sustainability and efficiency in bridging the water deficit, particularly in low- and middle-income countries, by providing pragmatic and affordable solutions to enable the circular use of water resources. Their modular and low-maintenance design makes them highly adaptable to areas lacking advanced infrastructure, while their operation reduces both greenhouse gas emissions and chemical dependency. These systems can be scaled to meet local needs, from household-level applications to community-scale installations. By treating wastewater at or near the point of generation, METs can facilitate water reuse for non-potable applications, including agricultural irrigation, industrial cooling, and groundwater recharge, thereby reducing freshwater demand.

**Figure 2:**
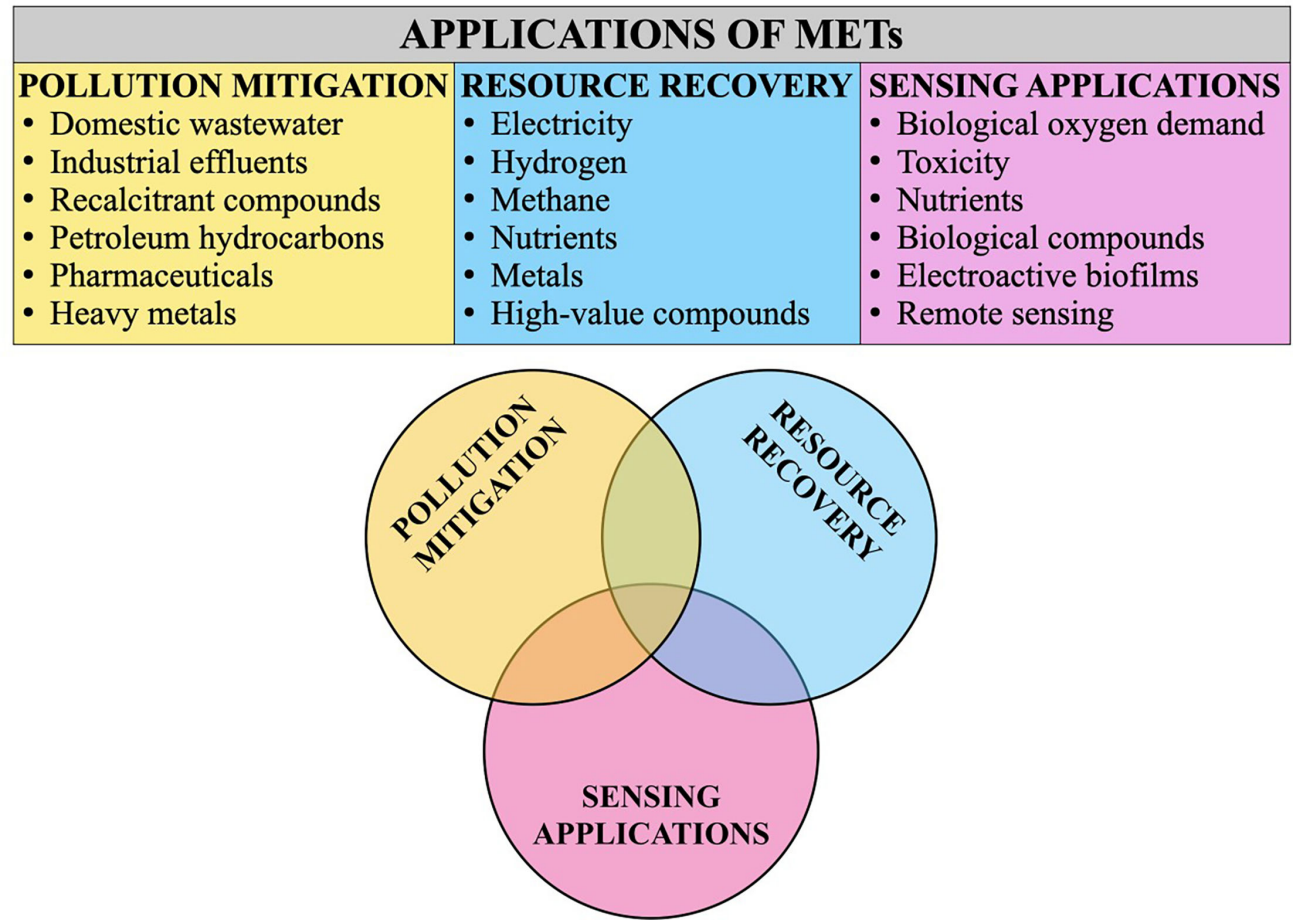
Applications of microbial electrochemical technologies.

METs leverage the natural metabolic activity of electrochemically active microorganisms to drive key electrochemical reactions. The metabolic diversity of these microbial consortia enables the utilization and complete mineralization of a broad spectrum of substrates to facilitate wastewater treatment [24], pollutant degradation [25] and the recovery of energy in the form of electricity, hydrogen, and methane; nutrients including nitrogen and phosphorus; chemicals such as acetate, propionate, butyrate, and other short-chain fatty acids; and metals from wastewater, thereby contributing to sustainable environmental management [26]. METs operate under anaerobic or microaerobic conditions, significantly reducing energy requirements. Moreover, configurations that enable electricity or hydrogen recovery can also achieve energy-positive operation. Direct electron transfer between microorganisms and electrodes allows METs to function as self-sustained biosensors, enabling real-time monitoring of water quality through measurable electrochemical signals [27,28]. Consequently, METs represent a promising pathway toward achieving energy-neutral and resource-recovering water treatment, contributing to the realization of SDGs related to clean water, sanitation, and climate resilience (Figure 2).

### Sustainability impact of METs

**Figure 3:**
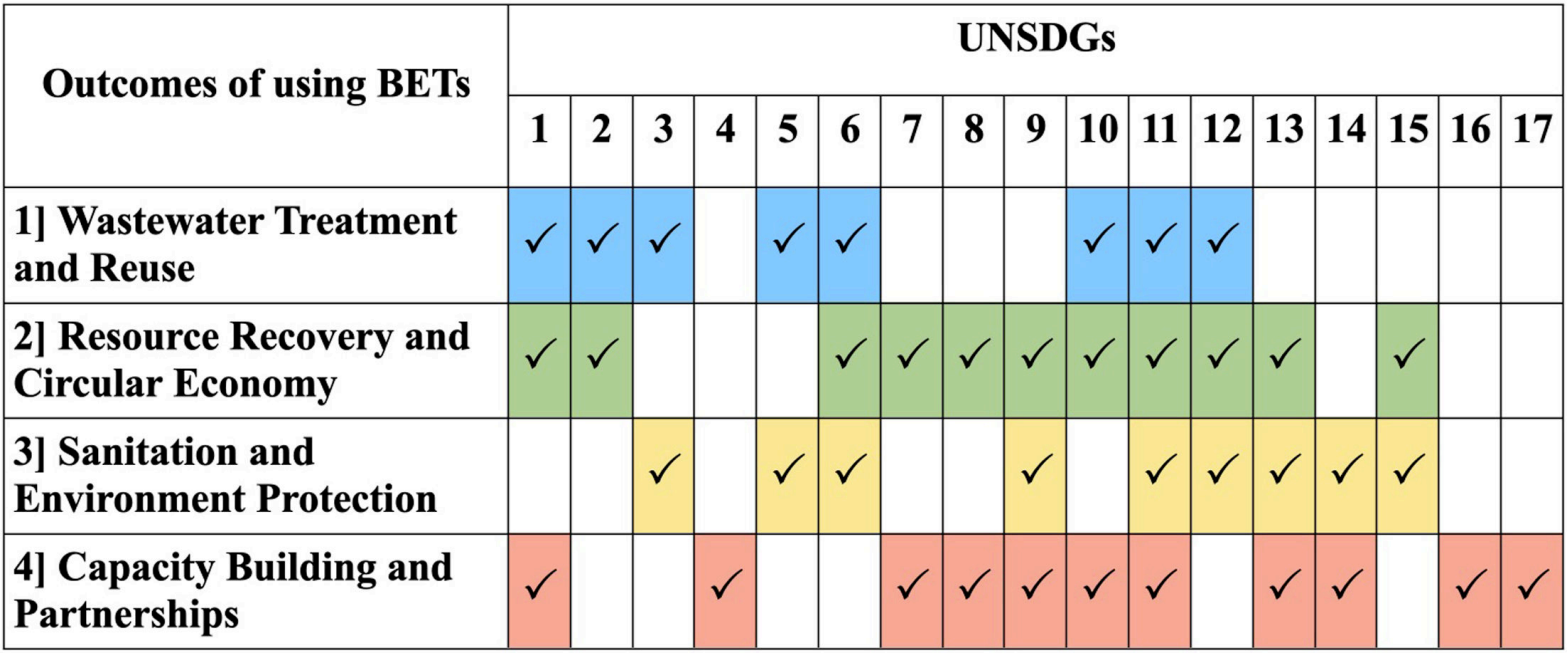
Mapping the outcomes of using METs with the UNSDGs

The use of METs contributes to the UNSDGs through several major outcomes. These outcomes broadly map to numerous specific UNSDGs (Figure 3). The key areas of contribution include:

1. Wastewater treatment and reuse: METs enable the design of need-based, modular, decentralized wastewater treatment systems suitable for remote areas. Increased wastewater treatment capacity improves water use efficiency by promoting reuse for non-potable applications in agriculture and industry. This can lead to more sustainable agricultural practices, potentially enhancing farmer income and quality of life. Improved access to clean water also has a positive impact on women and girls, who often bear the responsibility for water collection in many communities.
2. Resource recovery and circular economy: Recovery of renewable energy from wastewater makes treatment processes energy-efficient and sustainable. Organic wastes can be transformed into high-value products, stimulating a circular economy. Nutrient removal from wastewater helps mitigate eutrophication. The installation and maintenance of these systems require skilled labor, facilitating job creation, poverty alleviation, and economic growth.
3. Sanitation and environment protection: Wastewater surveillance enabled by METs can drive action towards sanitation and the prevention of waterborne diseases, especially in tropical, low-income countries. Tracking and removal of specific contaminants help industries meet regulatory discharge limits. Sensing devices for monitoring aquatic habitats enhance scientific knowledge and support policy-making for the protection, conservation, and restoration of water-related ecosystems. MET integration helps significantly minimize the carbon footprint and improve climate resilience.
4. Capacity building and partnerships: Promoting METs raises awareness and fosters a sense of responsibility regarding sustainable water management. It provides a practical understanding of real-world problems, encouraging critical thinking and problem-solving skills. Scaling up requires multidisciplinary research to reduce costs and improve performance, fostering interest in science, technology, engineering, and mathematics (STEM) education. Extensive networking among governments, non-governmental organizations (NGOs), and the private sector is essential for feasible implementation.

### Challenges and research priorities

While METs show significant promise for contributing to numerous UNSDG targets, improvement in efficiency is essential to translate the potential of METs into practical, scalable solutions for sustainable water management. Focused research, development, and collaborative efforts are essential to address challenges facing their widespread adoption.

One of the most significant challenges is that power density and treatment efficiency do not scale linearly with reactor size, mainly due to increased internal resistance and mass transfer limitations. METs require time to establish electroactive biofilms, and this delayed start-up reduces their flexibility for intermittent use and makes deployment difficult in decentralized systems where flow conditions often vary. Initial investment for METs can be significantly higher than conventional wastewater treatment systems, primarily due to the cost of materials and specialized reactor construction. Beyond scientific and engineering constraints, large-scale implementation is further limited by techno-economic barriers, including the high capital cost of corrosion-resistant electrodes and membrane materials, modest power densities, and relatively lower treatment rates compared with conventional energy-intensive systems [29]. Furthermore, concerns over the long-term stability and robustness of these systems under real-world, variable wastewater conditions hinder commercial uptake.

Enhanced biofilm formation [30] and efficient bacterial electron transfer hold the key to the performance of METs. The structure and activity of microbial communities constituting the biofilms at the anode have to be studied in greater detail to maintain consistency in electron transfer over extended time periods [31]. Electrode materials that serve as the interface for bacterial electron transfer may have to undergo more generational shifts to minimize losses and costs [32]. Standardization of key parameters and operating conditions is critical for performance benchmarking. Optimization of design and configuration must be pursued alongside scale-up operations to ensure cost-effectiveness [29]. Identifying and presenting common issues associated with long-term operation and maintenance as public challenges could encourage troubleshooters and accelerate solutions. Life cycle analyses of METs integrated with conventional wastewater treatment systems are needed to provide a clear picture of their overall environmental impact. Engineering interventions must be supported by scientific investigations to plug the knowledge gaps in our understanding of microbial electrochemical processes. As noted earlier, technological scalability and the development of appropriate regulatory frameworks remain significant hurdles.

The establishment of supportive regulatory frameworks and targeted policy measures is essential to overcome these challenges and to accelerate adoption. For METs to emerge as viable platforms for resource recovery and clean energy generation, regulatory policies should recognize treated wastewater, recovered bioenergy, and electrochemical by-products as legitimate products or value-added services rather than merely waste treatment outcomes [33]. Such recognition would enable monetization and access to incentives. Financial mechanisms such as tax credits, grants, and public–private partnerships can mitigate early deployment risks and encourage adoption. Establishing standards for performance, safety, and environmental impact will build confidence among stakeholders, while policies promoting resource recovery in wastewater systems can align infrastructure and regulations toward circular-economy goals. Finally, long-term monitoring and transparent data sharing, supported by regulators, will foster continual improvement and public trust [34] in large-scale MET implementation to address the UNSDGs.

## Conclusion

The close association between METs and the UNSDGs presents a potentially transformative path towards a sustainable future. Integrating METs into wastewater treatment processes exemplifies how technological innovation can simultaneously contribute to multiple UNSDG targets [35]. While progress has been made, collective efforts are required to accelerate the achievement of these goals. Alongside scientific advancements in addressing the technical and operational challenges of METs, creating widespread awareness of their immense potential is of critical importance to drive adoption and realize their full benefits for global sustainable development. The path forward requires a concerted effort to transition METs from innovative concepts to foundational solutions. This demands focused research on enhancing efficiency, developing advanced materials, and standardizing processes, but it also requires supportive policy and widespread awareness to drive adoption. By truly harnessing these bioelectrochemical systems, we can position METs as a cornerstone technology for the coming decades, fundamentally shifting our global water management paradigm from ‘treatment and disposal’ to ‘recovery and reuse’—creating a circular economy that simultaneously restores our planet’s water resources and powers our sustainable future.

## Abbreviations

METs: microbial electrochemical technologies
NGOs: non-governmental organizations
SDG: sustainable development goal
UNSDGs: United Nations Sustainable Development Goals
